# Predicting Hyperglycemia Among Patients Receiving Alpelisib Plus Fulvestrant for Metastatic Breast Cancer

**DOI:** 10.1093/oncolo/oyad024

**Published:** 2023-03-21

**Authors:** Xuan Ge, Carolyn E Behrendt, Susan E Yost, Niki Patel, Raynald Samoa, Daphne Stewart, Mina Sedrak, Sayeh Lavasani, James Waisman, Yuan Yuan, Joanne Mortimer

**Affiliations:** Department of Medical Oncology & Therapeutics Research, City of Hope National Medical Center, Duarte, CA, USA; Department of Computational and Quantitative Medicine, City of Hope National Medical Center, Duarte, CA, USA; Department of Medical Oncology & Therapeutics Research, City of Hope National Medical Center, Duarte, CA, USA; Department of Medical Oncology & Therapeutics Research, City of Hope National Medical Center, Duarte, CA, USA; Department of Diabetes and Endocrinology, City of Hope National Medical Center, Duarte, CA, USA; Department of Medical Oncology & Therapeutics Research, City of Hope National Medical Center, Duarte, CA, USA; Department of Medical Oncology & Therapeutics Research, City of Hope National Medical Center, Duarte, CA, USA; Department of Medical Oncology & Therapeutics Research, City of Hope National Medical Center, Duarte, CA, USA; Department of Medical Oncology & Therapeutics Research, City of Hope National Medical Center, Duarte, CA, USA; Department of Medical Oncology & Therapeutics Research, City of Hope National Medical Center, Duarte, CA, USA; Department of Medical Oncology & Therapeutics Research, City of Hope National Medical Center, Duarte, CA, USA

**Keywords:** hyperglycemia, alpelisib, fulvestrant, breast cancer, quality of life

## Abstract

**Background:**

Hyperglycemia is recognized as a common adverse event for patients receiving alpelisib but has been little studied outside of clinical trials. We report the frequency of alpelisib-associated hyperglycemia in a real-world setting and evaluate proposed risk factors.

**Patients and Methods:**

We retrospectively identified patients with PIK3CA-mutated, hormone receptor-positive, metastatic breast cancer who initiated treatment with alpelisib plus fulvestrant between August 2019 and December 2021. Ordinal logistic regression evaluated 5 characteristics (diabetes, prediabetes, body mass index [BMI], age, and Asian ancestry) as independent risk factors for ALP-associated hyperglycemia grades 2-4. Risk of error from multiple hypothesis testing was controlled using the false discovery rate method.

**Results:**

The study included *n* = 92 subjects, all but 1 female, mean age 59.9 (+11.9) years with 50% non-Hispanic White, 15% Hispanic/Latino, 13% Asian, 9% African/Black, and 13% other/unknown. In total 34% of patients had diabetes, 10% had pre-diabetes, and 56% had normoglycemia. Thirty-six percent were obese, 32% were overweight, 25% were normal weight, and 7% were lean. Frequency of grades 1-4 hyperglycemia in current subjects (64.1%) was similar to hyperglycemia reported in the SOLAR-1 trial (63.7%). Our subjects’ risk of grades 2-4 hyperglycemia was independently increased by pre-existing diabetes (Odds ratio 3.75, 95% CI, 1.40-10.01), pre-diabetes (6.22, 1.12-34.47), Asian ancestry (7.10, 1.75-28.84), and each unit of BMI above 20 (1.17, 1.07-1.28).

**Conclusion:**

While receiving alpelisib, patients of Asian ancestry, as well as patients with pre-existing hyperglycemia and/or BMI above 20, should be closely monitored for hyperglycemia. The mechanism underlying the current association of alpelisib-associated hyperglycemia with Asian ancestry is independent of BMI and merits further study. The high incidence of hyperglycemia resulted in a change in practice to include consultation with a diabetes nurse educator or endocrinologist at the start of alpelisib.

## Teaser

Hyperglycemia is a common adverse event for patients receiving alpelisib. This article reports the frequency of alpelisib-associated hyperglycemia in a real-world setting and evaluates proposed risk factors.

**Implications for Practice**

Findings from the current study suggest that patients with breast cancer with one or more risk factors (Asian ancestry, pre-existing diabetes or pre-diabetes, and/or BMI above 20) should be closely monitored for hyperglycemia while receiving alpelisib. Neither age, hypertension, nor hyperlipidemia increases the risk of this adverse reaction.

## Introduction

In May 2019, alpelisib (PIQRAY, Novartis Pharmaceuticals Corporation) was given FDA approval in combination with fulvestrant for postmenopausal women and men with recurrent/metastatic hormone receptor positive HER2-negative breast cancer patients who had a ­phosphatidylinositol-4,5-bisphosphate 3-kinase catalytic subunit alpha (PIK3CA) mutation. The approval was based on the SOLAR-1 trial which demonstrated that the addition of alpelisib to fulvestrant improved progression-free survival from 5.7 to 11 months.^1^ The inhibition of PI3Kα induces insulin resistance and results in hyperglycemia.^2^ The SOLAR-1 trial excluded patients with type 1 diabetes and those with uncontrolled type 2 defined as a fasting glucose of >140 mg/dL or glycosylated hemoglobin level of > 6.4%. The most common side effect in this trial was hyperglycemia which occurred in 63.7% of alpelisib-treated patients. Additional toxicities included diarrhea (57.7%), nausea (44.7%), decreased appetite (35.6%), and rash (35.6%).^1,3^ We observed the similar toxicity profile in Bylieve study.^4^

The study inclusion for SOLAR-1 was not overly restrictive but may not have represented the real-world population of women with breast cancer. In the developing world, 16% of women with metastatic breast cancer with diabetes were considered ineligible for SOLAR-1.^1^ We undertook a retrospective chart review study of women with metastatic hormone receptor positive breast cancer treated with fulvestrant and alpelisib to determine the incidence of hyperglycemia and to identify risk factors for its development.

## Methods

With a waiver of consent from the Institutional Review Board, data for the current study were abstracted retrospectively from the subjects’ medical charts. Subjects were patients at our institution who began treatment with alpelisib for advanced breast cancer between August 2019 and December 2021. Excluded were patients who dissented from to the use of their medical data in research.

Most BMI values available from the patient chart were measured after the start of treatment with alpelisib. Because the drug is known to cause decrease in body weight and because our risk factor analysis required BMI at pre-alpelisib baseline, we sought to estimate pre-alpelisib BMI from the patient’s post-alpelisib value. To do so, we modeled the extent to which recorded BMI varied by its timing after initiation of alpelisib. That generalized linear regression model specified a Poisson distribution, and was adjusted for all covariates that improved the model’s fit to the observed data and explored whether BMI’s slope over time was modified by any covariate. Using the slopes identified by the model, all BMI values obtained after alpelisib initiation were converted to their ­model-estimated ­pre-alpelisib value using the following formula. Specifically, for patients with prior diabetes or pre-diabetes, ­pre-alpelisib BMI was calculated as exp[log(BMI from chart) − log(days after alpelisib initiation × −0.036)], while for patients with normoglycemic history, pre-alpelisib BMI was calculated as exp[log(BMI from chart) − log(days after alpelisib initiation × −0.003)]. Subjects were classified by their pre-alpelisib BMI values into categories of lean, normal, overweight, and obese at baseline using the appropriate ­ancestry-specific cut-point standards (for Asian and non-Asian ancestries, respectively).^5,6^

The distribution of hyperglycemia among current subjects was compared to that reported in the SOLAR-1 trial^1^ using the Jonckheere-Terpstra test^7^ (appropriate for a 2-way table with an ordinal outcome, ie, grade of hyperglycemia).

The hypothesized risk factors for hyperglycemia were evaluated using ordinal logistic regression, a method appropriate for the ranked outcome under study. For that model, the Akaike Information Criterion was used to define the outcome categories. Specifically, it was most efficient to define the highest ranked outcome by combining the grade 3 cases with the few grade 4 cases. In addition, analyzing the few grade 1 cases as a separate outcome worsened the model’s fit. For clarity, the grade 1 cases were set aside rather than combined with normoglycemia. As a result, the model considered only mid- (grade 2) and high-level (grades 3-4) hyperglycemia in comparison to normoglycemia (the lowest-ranked outcome). For that regression model, it was found optimal to enter BMI as continuous variable having a minimum threshold of 20. No minimum threshold, categorization, or transformation of age was found to change the evident lack of association between age and hyperglycemia. The false discovery rate method^8^ was used to limit to below 5% the overall risk of error from evaluating 5 hypothesized risk factors for hyperglycemia on alpelisib.

## Results

### Patients

The study included *n* = 92 subjects, all but 1 female, mean age of 59.9 (+11.9) years, with 50% non-Hispanic White, 15% Hispanic/Latino, 13% Asian, 9% African/Black, and 13% other/unknown. In total 34% of patients had diabetes, 10% had pre-diabetes, and 56% had normoglycemia, 36% were obese, 32% were overweight, 25% were normal weight, and 7% were lean (Table 1). With a single exception, all subjects were female. Patients began alpelisib at mean age 59.9 (±11.9) years. Treatment was initiated in 2019 (*n* = 22), 2020 (*n* = 32), or 2021 (*n* = 37). At the time of chart review, 55/92 (60%) subjects had discontinued alpelisib (median 74, range 5-437, days on drug) and 37/92 (40%) were still receiving alpelisib (median 117, range 12-511, days on drug).

**Table 1.** Patient characteristics prior to initiating alpelisib (*N* = 92).

	*N* (%)
Age	
75-94 years	9 (9.8)
55-74 years	22 (23.9)
Less than 55 years	61 (66.3)
Body mass index^*^	
Obese	33 (35.9)
Overweight	29 (31.5)
Normal	23 (25.0)
Lean	7 (7.6)
Glycemic history	
Diabetes	31 (33.7)
Pre-diabetes only	9 (9.8)
Normoglycemia	52 (56.5)
Diagnosis of hypertension	
Present	49 (53.3)
Absent	43 (46.7)
Diagnosis of hyperlipidemia	
Present	38 (41.3)
Absent	54 (58.7)
Self-reported ancestry	
Asian	12 (13.0)
Hispanic/Latino	14 (15.2)
African/Black	8 (8.7)
Hawaiian/Pacific Islander	1 (1.1)
Other	2 (2.2)
Non-Hispanic White	46 (50.0)
Unknown/decline to state	9 (9.8)

^*^As described in the Methods, Results, and  Supplementary Table S1, for *n* = 64 subjects it was necessary to estimate the pre-alpelisib value of BMI from a post-alpelisib value in their medical chart. Lean was defined as BMI < 20, normal was defined as BMI 20 to <25 (BMI 20 to <23 for Asians), overweight was defined as BMI 25 to <30 (BMI 23 to <27.5 for Asians), and obese was defined as BMI ≥30 (BMI ≥27.5 for Asians).^5,6^

In this retrospective study, the BMI measured closest to initiation of alpelisib was recorded median of 49.5 days ­post-initiation (range 1255 days before to 383 days after initiation). According to a Poisson regression model of recorded BMI (adjusted for pre-existing hypertension, Asian ancestry, age, and prior hyperglycemia), the relationship between BMI and its post-alpelisib timing depended on the patient’s glycemic history prior to alpelisib (*P* < .05) (Supplementary Table S1). Specifically, in patients with pre-existing hyperglycemia, (log) BMI decreased significantly with (log) days elapsed since initiating alpelisib. In contrast, in patients with normoglycemic history, no significant decline in (log) BMI occurred post-alpelisib.

Post-initiation BMI values (from *n* = 64 patients, all female) were converted to their estimated pre-alpelisib value using the slopes from the model in Supplementary Table S1. Thus, for patients with prior history of hyperglycemia, the model estimated missing pre-alpelisib BMI values (*n* = 27) to be median +4.52 (range +1.11, +6.92) BMI units higher than the post-alpelisib observation, by which time the patients had lost an estimated −13.9% (range −5.6%, −19.2%) of their pre-alpelisib BMI. In contrast, previously normoglycemic patients had model-estimated pre-alpelisib BMI values (*n* = 37) that were scarcely higher (median +0.34, range +0.09, +0.77) than their post-alpelisib observation, corresponding to an estimated −1.4% (range −0.4%, −1.9%) decrease in BMI on alpelisib. By thus imputing pre-alpelisib BMI values for the *n* = 64 subjects whose BMI had been measured post-alpelisib, it was found that 16/64 (25%) patients shifted BMI category after initiating alpelisib: These patients included 1 of 23 normal weight pre-alpelisib who became lean, 8 of 29 overweight pre-alpelisib who became normal weight, and 9 of 33 obese pre-alpelisib who became overweight (*n* = 8) or normal weight (*n* = 1) after initiating alpelisib.

### Hyperglycemia

While receiving alpelisib, 59/92 (64.1%) current subjects developed hyperglycemia of any grade, similar to 181/284 (63.7%, no significant difference) observed among alpelisib recipients in the SOLAR-1 trial.^1^ Likewise, grade of hyperglycemia (Table 2) was not differently distributed in these 2 groups of patients (2-sided Jonckheere-Terpstra test, *P* = .66).

**Table 2.** Maximum glycemic outcome on alpelisib, by study.^9^

	Current study, *n* (%)	SOLAR-1 Trial alpelisib arm, *n* (%)
Grade 4	4 (4.4)	11 (3.9)
Grade 3	22 (23.9)	93 (32.7)
Grade 2	25 (27.2)	45 (15.8)
Grade 1	8 (8.7)	32 (11.3)
Normoglycemia only	33 (35.9)	103 (36.3)
Total	92	284

Turning to the risk factor analysis, multivariable modeling (Table 3) showed that risk for grades 2-4 hyperglycemia was independently increased by 4 of the 5 hypothesized risk factors: diabetes, pre-diabetes, Asian ancestry, and unit of ­pre-alpelisib BMI above 20. The magnitude of hyperglycemia risk associated with diabetes did not differ from that associated with pre-diabetes (comparison, *P* = .56). The association with BMI was independent of and unmodified by Asian ancestry. Exploratory analysis detected no univariable or multivariable association with preexisting hypertension or hyperlipidemia (data not shown).

**Table 3.** Multivariable evaluation of hypothesized risk factors for grades 2-4 hyperglycemia on alpelisib (*N* = 84).

Baseline characteristics	*N* subjects	Odds ratio (95% CI)	*P*
Glycemic history			
Diabetes	30	3.75 (1.40-10.01)	<.01^*^
Pre-diabetes only	8	6.22 (1.12-34.47)	.04^*^
Neither	46	1.00	
Asian ancestry			
Yes	11	7.10 (1.75-28.84)	.01^*^
No	73	1.00	<.001^*^
Per unit of BMI above 20	84	1.17 (1.07-1.28)	.62
Per year of age	84	1.01 (0.97-1.05)	

^*^The model’s overall false discovery rate remains below 5% when all 4 hypotheses with asterisk are accepted as statistically significant.

### Asian Ancestry

All patients who reported Asian ancestry were female and had Chinese (*n* = 8) or Filipino (*n* = 4) surnames. Their ages ranged from 36.5 to 82.1 years, their pre-alpelisib BMI ranged between 19.5 and 28.8, and 6 of the 12 had pre-existing diabetes. Grades 2-4 hyperglycemia developed in 9 patients of Asian ancestry, specifically the 6 patients with pre-existing diabetes, and 3 normoglycemic patients who were under age 65 and normal-weight, overweight, or obese, respectively.

## Discussion

We report our real-world experience with alpelisib in an unselected population of women with advanced PIK3A mutated hormone receptor positive breast cancer progressing on prior endocrine therapy. Of the 92 patients included in the analysis, 64.1% of whom developed hyperglycemia which was grade 3/4 in 28.3% which was similar to what was reported in the SOLAR-1 trial. In our analysis, grade 2+ hyperglycemia and grade 2+ rash tend to be early effects (median onset of 15 and 13 days, respectively), whereas grade 2+ gastrointestinal toxicity can begin at any time during treatment. The risk of hyperglycemia on alpelisib (especially grades 3-4) was associated with baseline glycemic status (diabetes > prediabetes > normoglycemia), body mass index (BMI) (obesity > overweight > normal weight), and advanced age (above age 75 years versus below). However, whether glycemic history, BMI, and age are independent risk factors for this AE remains to be determined. In contrast to hyperglycemia, rash on alpelisib was associated solely with prophylactic use of antihistamine, and gastrointestinal toxicity was possibly associated with advanced age.^9^ The primary objective of the current study was to retrospectively evaluate the independent significance of 5 hypothesized risk factors for alpelisib-associated hyperglycemia. Diabetes, prediabetes, BMI, and age were described from SOLAR-1,^1^ but Asian ancestry was not reported. Our data support the findings from SOLAR-1 that ­pre-existing hyperglycemia and BMI predispose patients to develop hyperglycemia on alpelisib. Our work demonstrates not only that these risk factors are independent, but also that risk from BMI begins after BMI 20 and rises incrementally, and diabetes and pre-diabetes confer similar degrees of risk.

This study is the first to identify Asian ancestry as a novel risk factor for alpelisib-associated hyperglycemia. Other chemotherapeutic agents have been found to cause greater toxicity in patients with East Asian ancestry compared to their Western patients.^10-12^

The possible biological mechanism underlying the current association between Asian ancestry and alpelisib-associated hyperglycemia remains to be elucidated. A heightened susceptibility to hyperglycemia has been observed among patients of Asian ancestry. In addition, an elevated prevalence of diabetes is present among patients of Filipino, Vietnamese, Korean, South Asian, or Japanese descent whose BMI is classified as normal or overweight using standard cut-points but actually corresponds to overweight or obese under the Asian-specific cut-points recommended by the World Health Organization.^5,6^ However, our multivariable analysis makes clear that the current association between Asian ancestry and alpelisib-associated hyperglycemia is independent of BMI, and thus cannot be explained by it. Moreover, our analysis detected no interaction between ancestry and BMI, indicating that the association between hyperglycemia and BMI does not differ for persons of Asian versus non-Asian ancestry. A potential explanation for the current association with Asian ancestry may involve genetic polymorphism in a cytochrome P450 enzyme. Investigating this possibility will require a larger multi-institutional sample of cancer patients.

An incidental finding of the current study is the novel observation that the decrease in BMI on alpelisib (from the decrease in body weight noted in the package insert^13^) is both time-dependent and modified by pre-existing glycemic status. This finding suggests that only those patients with ­pre-existing hyperglycemia will lose weight while on alpelisib.

Inhibition of PIK3A prevents glucose uptake in adipose tissue and skeletal muscle and activates hepatic glycogenolysis resulting in both and increase in glucose and release of insulin. The optimal management of alpelisib-induced hyperglycemia has yet to be determined. Use of metformin prior to starting and throughout alpelisib therapy has been reported with some success. Pioglitazone and sodium-glucose ­co-transporter 2 (SGLT2) inhibitors have been used in combination with metformin. The SGLT2 inhibitors prevent reabsorption of glucose in the kidney and preclinical data have found these agents to be superior to metformin in managing alpelisib hyperglycemia.^14^

The findings from this study led us to change the order set within the electronic record so that every patient who is started on alpelisib is also evaluated by a diabetes nurse educator or an endocrinologist.

The chief limitations of the current study are its limited sample size and retrospective design. Current sample size precluded our investigating whether ancestries other than Asian are associated with hyperglycemia and whether Asian ancestry is associated with other classes of AEs on alpelisib. The retrospective design precluded uniform assessment of BMI prior to initiation of alpelisib. However, our analysis was able to convert post-initiation BMI values to their estimated ­pre-alpelisib value. Future studies of alpelisib-associated hyperglycemia should be prospective in order to record the time to onset of each AE. The analysis of SOLAR-1 did plot time to hyperglycemia, and future studies should address whether current risk factors are associated with an accelerated onset.

## Conclusion

Breast cancer patients of Asian ancestry as well as patients with pre-existing hyperglycemia and/or BMI above 20 should be closely monitored for hyperglycemia while receiving alpelisib. Given the predictably high incidence of hyperglycemia with alpelisib, we routinely include consultations with a diabetic educator or endocrinologist.

## Supplementary Material

oyad024_suppl_Supplementary_Table_S1

## Data Availability

The data underlying this article will be shared on reasonable request to the corresponding author.
