# Supplementary material for: Predicting Hyperglycemia Among Patients Receiving Alpelisib Plus Fulvestrant for Metastatic Breast Cancer
Source: Oncologist. 2023 Mar 21;28(7):e488–92. doi: 10.1093/oncolo/oyad024 (PMC10322119; doi:10.1093/oncolo/oyad024)
Supplement: oyad024_suppl_Supplementary_Table_S1 [file oyad024_suppl_supplementary_table_s1.docx]

**Supplemental Table 1.** Poisson Regression Model of (log) Body Mass Index (N=92)

|  | Estimated (log) BMI  (95% Confidence Interval) | p |
| --- | --- | --- |
| (log) BMI of Referent Group*  Any Shift in Clinical Profile Prior to Alpelisib:  From Normoglycemic to Pre-Diabetes, or from Pre-Diabetes to Diabetes  From Normotension to Hypertension  From Non-Hispanic White to Asian Ancestry  From Age Below 60 to Age 60 and Older  Shift with Time:  Per (log) Days Since Initiation of Alpelisib  By Prior Glycemic Status  If Hyperglycemic Prior to Alpelisib  If Normoglycemic Prior to Alpelisib | 3.26 (3.16, 3.36)  +0.14 (+0.06, +0.23)  +0.12 (+0.04, +0.21)  -0.14 (-0.27, -0.01)  -0.11 (-0.20, -0.03)  -0.036 (-0.061, -0.011)  -0.003 (-0.027, +0.021) | <0.001  0.003  0.029  0.008  0.047 |

* The model’s referent group consists of patients who are non-Hispanic white, age below 60 years, normoglycemic and non-hypertensive at baseline, whose BMI was measured prior to initiation of alpelisib.
